# Treatment practices and survival outcomes for *IDH*-wildtype glioblastoma patients according to *MGMT* promoter methylation status: insights from the U.S. National Cancer Database

**DOI:** 10.1007/s11060-025-04952-y

**Published:** 2025-02-05

**Authors:** John Pham, David J. Cote, Keiko Kang, Robert G. Briggs, David Gomez, Apurva Prasad, Sindhu Daggupati, Jonathan Sisti, Frances Chow, Frank Attenello, Clark C. Chen, Gabriel Zada

**Affiliations:** 1 Department of Neurosurgery, The Warren Alpert Medical School of Brown University, Providence, RI 02903 USA; 2 Department of Neurosurgery, University of Southern California Keck School of Medicine, Los Angeles, CA 90033 USA

**Keywords:** Glioblastoma, *IDH* wildtype, *MGMT*, Treatment practices, Survival outcomes

## Abstract

**Purpose:**

Methylation of the O^6^-methylguanine-DNA methyltransferase (*MGMT*) promoter is an important prognostic marker in glioblastoma (GBM); however, its implementation in clinical practice remains understudied. Here, we assessed the prevalence of *MGMT* methylation status among GBM patients in the United States. Additionally, we evaluated treatment practices and survival outcomes of GBM patients according to *MGMT* promoter methylation status.

**Methods:**

The National Cancer Database was queried to identify all adult U.S. patients (≥ 18 years) diagnosed with *IDH*-wildtype GBM between 2018 and 2020. Treatment regimen was grouped into no chemotherapy and no radiotherapy, chemotherapy alone (without radiotherapy), radiotherapy alone (without chemotherapy), and chemoradiotherapy (chemotherapy and radiotherapy). Survival data were analyzed using Kaplan-Meier survival curves, log-rank tests, and multivariable Cox proportional hazard modeling.

**Results:**

A total of 20,734 patients were included, of whom 6,404 (30.9%) had *MGMT*-methylated GBM, 9,065 (43.7%) had *MGMT*-unmethylated tumors, and 5,265 (25.4%) had unknown methylation status. The median and three-year overall survival were 12.4 months and 15.5%, respectively, for the entire cohort (16.4 months and 23.9% for *MGMT*-methylated patients and 11.8 months and 9.8% for *MGMT*-unmethylated patients, *p* < 0.001). Chemoradiotherapy was less commonly used for elderly (≥ 70 years, 58.5%) than non-elderly (< 70 years, 79.2%) patients. Among elderly patients, radiotherapy alone was more commonly administered than chemotherapy alone for patients with *MGMT*-unmethylated tumors (11.2% vs. 2.1%) and *MGMT*-methylated tumors (6.6% vs. 3.9%). However, chemotherapy alone was associated with a lower mortality risk (HR 0.71, 95% CI 0.51–0.99, *p* = 0.04) than radiotherapy alone for elderly patients with *MGMT*-methylated tumors, while chemotherapy alone was associated with a higher mortality risk (HR 1.63, 95% CI 1.09–2.44, *p* = 0.02) than radiotherapy alone for elderly patients with *MGMT*-unmethylated tumors. Patients who were elderly, uninsured, insured through Medicaid, lived in zip codes with lower median education levels, or received care at non-academic programs were less likely to undergo *MGMT* testing.

**Conclusion:**

A high proportion of GBM patients in the United States undergo *MGMT* promoter testing, though significant sociodemographic disparities exist. While there was a decrease in chemoradiotherapy use with increasing age, radiotherapy alone was more commonly administered to elderly patients than chemotherapy alone irrespective of *MGMT* promoter methylation status.

**Supplementary Information:**

The online version contains supplementary material available at 10.1007/s11060-025-04952-y.

## Introduction

Glioblastoma (GBM) is the most common primary malignant brain tumor in adults, with an annual incidence of approximately 2 per 100,000 population [1]. First line treatment includes maximal safe surgical debulking followed by a combination of radiotherapy (RT) and chemotherapy (CT) with the alkylating agent temozolomide (TMZ) [2]. The O^6^-methylguanine-DNA methyltransferase (*MGMT*) gene encodes a DNA repair enzyme that removes methyl moieties from the O^6^-position of guanine, an important site of DNA alkylation, and therefore, counteracts the cytotoxic effects of TMZ [3–4]. Epigenetic silencing of the *MGMT* gene promoter via DNA methylation is highly predictive of greater overall and progression free survival with alkylating agents [5–7].

The modern management of intracranial gliomas relies on molecular profiling, not only for accurate diagnosis, but increasingly to inform treatment decisions [8–9]. Elderly patients, or patients with poor functional status, may not tolerate treatment with multimodal therapy. According to Society for Neuro-Oncology (SNO) and European Association of Neuro-Oncology (EANO) guidelines, treatment decisions for patients not eligible for combined chemoradiotherapy (CRT) should be based on *MGMT* promoter methylation status, with TMZ preferred for *MGMT*-methylated patients and RT preferred for *MGMT*-unmethylated patients, based on the results of the NOA-08 trial [8–11]. While most non-elderly patients are offered TMZ regardless of *MGMT* promoter methylation status due to a lack of effective alternative treatments, *MGMT* testing is still important to use as a prognostic marker and for stratifying patients in clinical trials [12].

Currently, guidelines from the National Comprehensive Cancer Network (NCCN) recommend *MGMT* testing for all patients with high grade glioma [13]. However, considerable challenges hinder reliable *MGMT* testing, such as a lack of consensus on cutoffs for categorizing methylation status, an absence of standardized testing protocols, and the amount and quality of DNA samples required for testing [14]. Consequently, the degree to which *MGMT* promoter testing is implemented in clinical practice remains understudied. In this study, we assessed the prevalence of *MGMT* testing among patients recently diagnosed with GBM in the United States. Additionally, we evaluated the treatment patterns and survival outcomes of patients by *MGMT* promoter methylation status.

## Methods

### Data source

Datasets from the 2018 to 2020 National Cancer Database (NCDB) were queried. The analysis was restricted to years 2018–2020 because the NCDB began incorporating brain molecular markers in 2018. As a collaboration between the American Cancer Society and Commission on Cancer (CoC) of the American College of Surgeons (ACS), the NCDB collects data from over 1,500 hospitals and contains > 85% of new primary brain tumor diagnoses in the United States [15]. This study was exempt from the primary author’s institutional review board approval because the NCDB contains de-identified data, for which consenting was not applicable.

### Patient population

All adult patients (≥ 18 years) diagnosed with grade 4 isocitrate dehydrogenase (*IDH*)-wildtype GBM (International Classification of Diseases for Oncology, Third Edition codes 9400, 9401, or 9440, with Brain Molecular Marker site-specific data item #3816 values of 2, 4, or 5) of the brain (primary site codes C71.0-C71.9) were included [16]. Tumor grade was confirmed with the pathological grade data item (derived from surgical resection) or the clinical grade data item (derived from biopsy). Exclusion criteria included patients diagnosed without microscopic confirmation or patients with more than one primary cancer.

### Study variables and primary outcome

Baseline sociodemographic, clinical, pathological, and treatment information were abstracted for all patients. Sociodemographics consisted of age at diagnosis, sex, race, ethnicity, median income and education levels determined from the patient’s zip code of residence, insurance type, facility type, facility location, and distance to provider. Clinical information included Charlson/Deyo comorbidity scores, tumor size, and tumor primary site. Tumor size in the NCDB represents the maximum 2-dimensional diameter of the tumor. Pathological information included methylation status of the *MGMT* promoter, which was categorized into unmethylated (code 0), methylated (codes 1, 2, or 3), and unknown (codes 7, 8, 9, or missing) based on site-specific data item #3889 [16]. Descriptions of methylation codes used to categorize patients can be found in Supplementary Table S1. Treatment information consisted of extent of resection categorized as biopsy (surgical code 0), subtotal resection (STR, surgical codes 20, 21, and 40), and gross total resection (GTR, surgical codes 30 and 55), and treatment regimen (no RT and no CT, RT alone, CT alone, or CRT). All treatment variables reflect the first course of treatment, defined as occurring before disease progression or recurrence. The primary outcome was overall survival (OS), measured from the date of diagnosis to the date of death or most recent follow-up. Patients still alive at the end of follow-up were censored.

### Statistical analysis

Variables were summarized as means with standard deviations (SD), medians with interquartile ranges (IQR), or counts with percentages. One-way ANOVA or Kruskal-Wallis tests were used to compare associations between continuous variables, while Chi-square tests were used to compare associations between categorical variables. 1-year, 3-year, and median OS were calculated using Kaplan-Meier methods, and differences in OS by extent of resection, treatment regimen, or *MGMT* promoter methylation status were compared with log-rank tests. Multivariable Cox proportional hazard models were constructed to evaluate the association between extent of resection and treatment regimen with all-cause mortality. The results were presented as hazard ratios (HR) with 95% confidence intervals (CI). The likelihood of undergoing *MGMT* promoter testing was assessed using a multivariable logistic regression model. The results were presented as odds ratios (OR) with 95% CI. All statistical analyses were performed using SPSS, version 28 (IBM Corp., Armonk, NY). All tests were two-sided, and statistical significance was accepted at the *p* < 0.05 level.

## Results

### Patient population and demographics

A total of 20,734 patients met inclusion criteria, of whom 6,404 (30.9%) had *MGMT*-methylated tumors, 9,065 (43.7%) had *MGMT*-unmethylated tumors, and 5,265 (25.4%) had unknown *MGMT* methylation status. With a median (IQR) age of 64 (56–71) years, the cohort consisted of 12,272 males (59.2%) and 18,286 White patients (89.2%). The majority of patients had a Charlson-Deyo comorbidity score of 0 (71.4%) and resided in metropolitan areas (84.6%). A plurality of patients held Medicare (45.3%) and received treatment at academic programs (48.3%). The median (IQR) tumor diameter of the entire cohort was 44 (32–55) millimeters. Comparisons of baseline patient characteristics grouped by *MGMT* methylation status are shown in Table 1.

**Table 1:** Baseline clinical and demographic characteristics of patients diagnosed with glioblastoma, *IDH*-wildtype from the NCDB by *MGMT* promoter methylation status

	No. (%)				
Characteristics	Total	*MGMT* Methylated	*MGMT* Unmethylated	*MGMT* Unknown	p value^*^
	(*n* = 20,734)	(*n* = 6,404)	(*n* = 9,065)	(*n* = 5,265)	
**Age**,** median (IQR)**,** years**	64 (56–71)	65 (57–72)	63 (55–70)	65 (56–72)	*p* < 0.001
**Age category**					*p* < 0.001
≤ 44	1,451 (7.0)	368 (5.7)	692 (7.6)	391 (7.4)	
45–64	9,188 (44.3)	2,759 (43.1)	4,249 (46.9)	2,180 (41.4)	
≥ 65	10,095 (48.7)	3,277 (51.2)	4,124 (45.5)	2,694 (51.2)	
**Sex**					*p* < 0.001
Males	12,272 (59.2)	3,468 (54.2)	5,676 (62.6)	3,128 (59.4)	
Females	8,462 (40.8)	2,936 (45.8)	3,389 (37.4)	2,137 (40.6)	
**Race** ^**1**^					*p* < 0.001
White	18,286 (89.2)	5,705 (90.0)	8,037 (89.7)	4,544 (87.6)	
Black	1,300 (6.3)	367 (5.8)	541 (6.0)	392 (7.6)	
Asian	554 (2.7)	162 (2.6)	254 (2.8)	138 (2.7)	
Other	352 (1.7)	106 (1.7)	132 (1.5)	114 (2.2)	
**Ethnicity**					*p* < 0.001
Non-Hispanic	19,000 (93.4)	5,948 (94.4)	8,328 (93.6)	4,724 (91.7)	
Hispanic	1,352 (6.6)	354 (5.6)	573 (6.4)	425 (8.3)	
**Charlson/Deyo Comorbidity Score Index**					*p* = 0.289
0	14,799 (71.4)	4,500 (70.3)	6,512 (71.8)	3,787 (71.9)	
1	3,161 (15.2)	1,031 (16.1)	1,339 (14.8)	791 (15.0)	
2	1,579 (7.6)	491 (7.7)	690 (7.6)	398 (7.6)	
3	1,195 (5.8)	382 (6.0)	524 (5.8)	289 (5.5)	
**Income**					*p* < 0.001
≥$63,333	7,488 (43.0)	2,386 (44.6)	3,391 (44.8)	1,711 (38.0)	
$50,354-$63,332	4,228 (24.3)	1,312 (24.5)	1,837 (24.3)	1,079 (24.0)	
$40,227-$50,353	3,424 (19.6)	1,038 (19.4)	1,446 (19.1)	940 (20.9)	
<$40,227	2,291 (13.1)	617 (11.5)	899 (11.9)	775 (17.2)	
**Education**,** % without HSD**					*p* < 0.001
< 5.0%	4,660 (26.7)	1,488 (27.7)	2,158 (28.4)	1,014 (22.5)	
5.0-9.0%	5,368 (30.7)	1,689 (31.5)	2,354 (31.0)	1,325 (29.4)	
9.1-15.2%	4,493 (25.7)	1,362 (25.4)	1,926 (25.4)	1,205 (26.7)	
≥ 15.3%	2,950 (16.9)	824 (15.4)	1,156 (15.2)	970 (21.5)	
**Geographic Region**					*p* = 0.150
Metropolitan	16,863 (84.6)	5,182 (84.4)	7,399 (84.9)	4,282 (84.2)	
Urban	2,752 (13.8)	843 (13.7)	1,195 (13.7)	714 (14.0)	
Rural	317 (1.6)	113 (1.8)	117 (1.3)	87 (1.7)	
**Insurance Status**					*p* < 0.001
Private	8,505 (41.5)	2,496 (39.4)	4,024 (44.9)	1,985 (38.2)	
Medicare	9,287 (45.3)	3,094 (48.8)	3,790 (42.3)	2,403 (46.3)	
Medicaid	1,615 (7.9)	473 (7.5)	690 (7.7)	452 (8.7)	
Other government	512 (2.5)	131 (2.1)	228 (2.5)	153 (2.9)	
Not insured	568 (2.8)	144 (2.3)	222 (2.5)	202 (3.9)	
**Facility Type**					*p* < 0.001
Academic	9,587 (48.3)	3,055 (49.5)	4,332 (50.0)	2,200 (43.8)	
Non-Academic	10,276 (51.7)	3,121 (50.5)	4,334 (50.0)	2,821 (56.2)	
**Facility location**					*p* < 0.001
West	3,697 (18.6)	1,302 (21.1)	1,662 (19.2)	733 (14.6)	
Midwest	4,881 (24.6)	1,528 (24.7)	2,236 (25.8)	1,117 (22.2)	
South	6,892 (34.7)	1,880 (30.4)	2,631 (30.4)	2,381 (47.4)	
Northeast	4,393 (22.1)	1,466 (23.7)	2,137 (24.7)	790 (15.7)	
**Distance to provider**, mean (SD), miles	3.40 (2.47)	3.38 (2.46)	3.37 (2.46)	3.49 (2.50)	*p* = 0.027
**Tumor Diameter**, median (IQR), mm	44 (32–55)	44 (32–55)	44 (32–55)	44 (31–56)	*p* = 0.784
**Tumor Diameter Category**					*p* = 0.035
< 25 mm	2,265 (13.2)	694 (12.9)	987 (13.0)	584 (14.0)	
25–49 mm	8,319 (48.6)	2,607 (48.6)	3,732 (49.1)	1,980 (47.5)	
50–99 mm	6,467 (37.8)	2,047 (38.2)	2,843 (37.4)	1,577 (37.8)	
≥ 100 mm	75 (0.4)	14 (0.3)	33 (0.4)	28 (0.7)	
**Primary Site**					*p* < 0.001
Cerebrum	775 (3.7)	229 (3.6)	325 (3.6)	221 (4.2)	
Frontal lobe	5,924 (28.6)	1,878 (29.3)	2,545 (28.1)	1,501 (28.5)	
Temporal lobe	5,328 (25.7)	1,621 (25.3)	2,409 (26.6)	1,298 (24.7)	
Parietal lobe	3,187 (15.4)	991 (15.5)	1,397 (15.4)	799 (15.2)	
Occipital lobe	757 (3.7)	228 (3.6)	348 (3.8)	181 (3.4)	
Ventricles	75 (0.4)	25 (0.4)	28 (0.3)	22 (0.4)	
Cerebellum	132 (0.6)	36 (0.6)	53 (0.6)	43 (0.8)	
Brainstem	70 (0.3)	11 (0.2)	35 (0.4)	24 (0.5)	
Overlapping	2,745 (13.2)	883 (13.8)	1,201 (13.2)	661 (12.6)	
Brain, Unspecified	1,741 (8.4)	502 (7.8)	724 (8.0)	515 (9.8)	

Abbreviations: No., number; IQR, interquartile range; SD, standard deviation; HSD, high school diploma; mm millimeter

^1^Other races includes American Indian, Aleutian, Eskimo, Micronesian, Chamorran, Guamanian, Polynesian, Tahitian, Samoan, Tongan, Melanesian, Fiji Islander, and New Guinean

^*^Variables with statistical significance are shown in bold

### Predictors of receiving *MGMT* testing

On multivariable logistic regression, age ≥ 65 (OR = 0.76, 95% CI 0.60–0.97) and residence in zip codes in the lowest (OR 0.71, 95% CI 0.62–0.82) and second lowest quartiles (OR 0.89, 95% CI 0.78–0.99) of education were associated with a decreased likelihood of receiving *MGMT* diagnostic testing (Table 2). Additionally, treatment at non-academic centers (OR 0.73, 95% CI 0.68–0.79) and hospital location in the Midwest (OR 0.76, 95% CI 0.67–0.86) and the South (OR 0.47, 95% CI 0.42–0.53) were significantly associated with lower odds of receiving *MGMT* testing. Compared to patients with private insurance, patients with Medicaid (OR 0.85, 95% CI 0.73–0.99) and no insurance (OR 0.72, 95% CI 0.58–0.89) were less likely to receive *MGMT* testing.

**Table 2:** Multivariable logistic regression identifying predictors of receiving *MGMT* testing

Characteristics	Odds ratio (95% CI)^1^	*p*-value^*^
**Age category**		*p* < 0.001
≤ 44	Reference	
45–64	0.976 (0.778–1.224)	*p* = 0.834
≥ 65	0.759 (0.595–0.968)	*p* = 0.026
**Sex**		
Males	Reference	
Females	1.044 (0.968–1.125)	*p* = 0.266
**Race** ^**2**^		*p* = 0.043
White	Reference	
Black	0.896 (0.772–1.041)	*p* = 0.152
Asian	0.897 (0.711–1.133)	*p* = 0.362
Other	0.723 (0.555–0.942)	*p* = 0.016
**Ethnicity**		
Non-Hispanic	Reference	
Hispanic	0.869 (0.747–1.011)	*p* = 0.069
**Charlson/Deyo Comorbidity Score Index**		*p* = 0.187
0	Reference	
1	1.082 (0.976-1.200)	*p* = 0.136
2	1.034 (0.900-1.189)	*p* = 0.637
3	1.156 (0.986–1.356)	*p* = 0.075
**Income**		*p* = 0.126
≥$63,333	Reference	
$50,354-$63,332	1.012 (0.912–1.124)	*p* = 0.818
$40,227-$50,353	1.045 (0.925–1.179)	*p* = 0.479
<$40,227	0.892 (0.770–1.034)	*p* = 0.128
**Education**,** % without HSD**		*p* < 0.001
< 5.0%	Reference	
5.0-9.0%	0.922 (0.829–1.026)	*p* = 0.137
9.1-15.2%	0.885 (0.784–0.999)	*p* = 0.048
≥ 15.3%	0.712 (0.615–0.824)	*p* < 0.001
**Geographic Region**		*p* = 0.467
Metropolitan	Reference	
Urban	1.043 (0.930–1.170)	*p* = 0.471
Rural	1.177 (0.875–1.581)	*p* = 0.281
**Insurance Status**		*p* = 0.002
Private	Reference	
Medicare	1.077 (0.957–1.213)	*p* = 0.218
Medicaid	0.852 (0.734–0.988)	*p* = 0.034
Other government	0.897 (0.707–1.140)	*p* = 0.375
Not insured	0.720 (0.580–0.893)	*p* = 0.003
**Facility Type**		
Academic	Reference	
Non-Academic	0.730 (0.676–0.787)	*p* < 0.001
**Facility location**		*p* < 0.001
West	Reference	
Midwest	0.761 (0.673–0.860)	*p* < 0.001
South	0.474 (0.424–0.529)	*p* < 0.001
Northeast	0.981 (0.862–1.118)	*p* = 0.776

Abbreviations: CI, confidence interval; HSD, high school diploma

^1^ Adjusted for age, sex, race, ethnicity, Charlson/Deyo comorbidity score, income, education, geographic region, insurance status, facility type, and facility location

^2^ Other races include American Indian, Aleutian, Eskimo, Micronesian, Chamorran, Guamanian, Polynesian, Tahitian, Samoan, Tongan, Melanesian, Fiji Islander, and New Guinean

^*^ Variables with statistical significance are shown in bold

### Treatment practices by *MGMT* methylation status among the entire cohort and stratified by age

Compared to patients with *MGMT*-methylated tumors, a larger, but not statistically significant proportion of patients with *MGMT*-unmethylated tumors underwent gross total resection in the overall cohort (42.9% vs. 41.7%) (Table 3). RT alone was more commonly administered than CT alone for patients with *MGMT*-unmethylated tumors (6.8% vs. 1.6%) and patients with *MGMT*-methylated tumors (4.2% vs. 2.7%). The majority of patients with *MGMT*-methylated tumors (76.7%) and *MGMT*-unmethylated tumors (74.5%) received CRT.

**Table 3:** Treatment patterns of patients diagnosed with glioblastoma, *IDH*-wildtype by *MGMT* promoter methylation status among the entire cohort and stratified by age

	No. (%)			
Entire Cohort	Total	*MGMT* Methylated	*MGMT* Unmethylated	p-value^*^
	(*n* = 20,734)	(*n* = 6,404)	(*n* = 9,065)	
**Extent of Resection**				*p* = 0.054
Biopsy	3,615 (17.6)	1,043 (16.4)	1,353 (15.0)	
Subtotal	8,482 (41.3)	2,659 (41.9)	3,786 (42.1)	
Gross total	8,465 (41.2)	2,650 (41.7)	3,860 (42.9)	
**Treatment Regimen**				*p* < 0.001
No radiation or chemotherapy	3,908 (19.3)	1,029 (16.5)	1,521 (17.2)	
Radiation	1,158 (5.7)	264 (4.2)	601 (6.8)	
Chemotherapy	446 (2.2)	166 (2.7)	139 (1.6)	
Chemoradiotherapy	14,695 (72.7)	4,796 (76.7)	6,597 (74.5)	
**Age < 70**				
**Extent of Resection**				*p* = 0.593
Biopsy	2,134 (15.1)	585 (13.8)	864 (13.3)	
Subtotal	5,892 (41.7)	1,802 (42.6)	2,741 (42.3)	
Gross total	6,111 (43.2)	1,839 (43.5)	2,879 (44.4)	
**Treatment Regimen**				*p* < 0.001
No radiation or chemotherapy	2,011 (14.5)	494 (11.9)	827 (12.9)	
Radiation	610 (4.4)	126 (3.0)	324 (5.1)	
Chemotherapy	272 (2.0)	84 (2.0)	88 (1.4)	
Chemoradiotherapy	11,005 (79.2)	3,455 (83.1)	5,150 (80.6)	
**Age ≥ 70**				
**Extent of Resection**				*p* = 0.208
Biopsy	1,481 (23.1)	458 (21.5)	489 (19.4)	
Subtotal	2,590 (40.3)	857 (40.3)	1,045 (41.6)	
Gross total	2,354 (36.6)	811 (38.1)	981 (39.0)	
**Treatment Regimen**				*p* < 0.001
No radiation or chemotherapy	1,897 (30.1)	535 (25.5)	694 (28.1)	
Radiation	548 (8.7)	138 (6.6)	277 (11.2)	
Chemotherapy	174 (2.8)	82 (3.9)	51 (2.1)	
Chemoradiotherapy	3,690 (58.5)	1,341 (64.0)	1,447 (58.6)	

Abbreviation: No., number

^*^Variables with statistical significance are shown in bold

CRT was less commonly used for elderly (≥ 70 years, 58.5%) than non-elderly (< 70 years, 79.2%) patients. A higher proportion of elderly patients received no RT or CT (30.1%) than non-elderly patients (14.5%). Among elderly patients, RT alone was more commonly administered than CT alone for patients with *MGMT*-unmethylated tumors (11.2% vs. 2.1%) and *MGMT*-methylated tumors (6.6% vs. 3.9%). No statistically significant differences were observed in extent of tumor resection based on *MGMT* promoter methylation status in the elderly (*p* = 0.208) and non-elderly (*p* = 0.593) cohorts.

### Overall survival by *MGMT* methylation status among the entire cohort

**Fig. 1:**
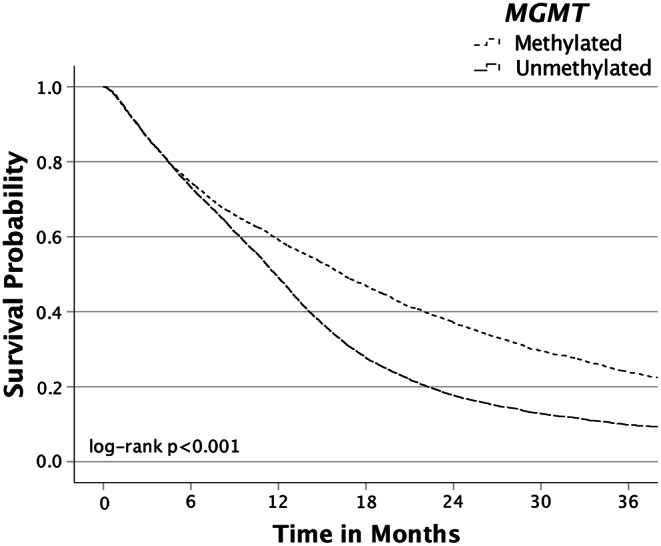
Kaplan-Meier survival curves in patients diagnosed with glioblastoma, *IDH*-wildtype from the NCDB by *MGMT* promoter methylation status

The 1-year, 3-year, and median OS of the entire cohort were 51.2%, 15.5%, and 12.4 months, respectively (Table 4). Patients with *MGMT*-methylated tumors had superior 1-year, 3-year, and median OS (59.1%, 23.9%, and 16.4 months) when compared to patients with *MGMT*-unmethylated tumors (49.1%, 9.8%, and 11.8 months) (*p* < 0.001) (Table 4; Fig. 1). GTR was associated with improved median OS (15.9 months) compared to STR (11.9 months) and biopsy (5.2 months), with similar results among patients with *MGMT*-methylated or *MGMT*-unmethylated tumors. CRT was associated with longer median OS (15.4 months) compared to RT alone (6.9 months), CT alone (7.1 months), and no RT or CT at all (2.5 months), with similar results after stratifying patients by *MGMT* promoter methylation status.

**Table 4:** Survival analyses of patients diagnosed with glioblastoma, *IDH*-wildtype among the entire cohort and stratified by *MGMT* promoter methylation status

	1-year OS	3-year OS	Median OS (95% CI)	*p*-value^*^	Hazard Ratio (95% CI)^1^	*p*-value^*^
**Entire Cohort**	51.2%	15.5%	12.4 (12.2–12.6)			
Extent of Resection				*p* < 0.001		*p* < 0.001
Biopsy	26.8%	6.0%	5.2 (4.9–5.5)		Reference	
Subtotal	49.70%	14.5%	11.9 (11.6–12.2)		0.627 (0.596–0.659)	*p* < 0.001
Gross total	62.8%	20.2%	15.9 (15.5–16.2)		0.486 (0.486–0.511)	*p* < 0.001
Treatment Regimen				*p* < 0.001		*p* < 0.001
No radiation or chemotherapy	16.6%	7.0%	2.5 (2.4–2.6)		1.701 (1.567–1.846)	*p* < 0.001
Radiation	29.0%	6.9%	6.9 (6.3–7.5)		Reference	
Chemotherapy	33.8%	10.3%	7.1 (6.1–8.1)		0.924 (0.804–1.062)	*p* = 0.267
Chemoradiotherapy	62.2%	18.2%	15.4 (15.2–15.7)		0.553 (0.512–0.596)	*p* < 0.001
***MGMT*** **Methylated**	59.1%	23.9%	16.4 (15.7–17.0)			
Extent of Resection				*p* < 0.001		*p* < 0.001
Biopsy	34.4%	7.9%	6.1 (5.4–6.8)		Reference	
Subtotal	57.2%	22.6%	15.6 (14.6–16.6)		0.604 (0.550–0.664)	*p* < 0.001
Gross total	70.4%	31.1%	21.8 (20.5–23.0)		0.475 (0.431–0.523)	*p* < 0.001
Treatment Regimen				*p* < 0.001		*p* < 0.001
No radiation or chemotherapy	15.7%	7.2%	2.4 (2.2–2.6)		1.657 (1.400-1.961)	*p* < 0.001
Radiation	28.70%	9.3%	6.3 (5.2–7.4)		Reference	
Chemotherapy	31.5%	10.5%	7.2 (5.4–9.1)		0.843 (0.661–1.075)	*p* = 0.168
Chemoradiotherapy	70.7%	28.4%	20.7 (19.9–21.4)		0.433 (0.370–0.507)	*p* < 0.001
***MGMT*** **Unmethylated**	49.1%	9.8%	11.8 (11.6–12.0)			
Extent of Resection				*p* < 0.001		*p* < 0.001
Biopsy	24.0%	4.7%	5.4 (4.9–5.9)		Reference	
Subtotal	46.0%	8.4%	11.1 (10.7–11.4)		0.686 (0.634–0.743)	*p* < 0.001
Gross total	60.4%	12.9%	14.3 (14.0-14.7)		0.503 (0.464–0.545)	*p* < 0.001
**Treatment Regimen**				*p* < 0.001		*p* < 0.001
No radiation or chemotherapy	17.7%	7.0%	2.6 (2.4–2.8)		1.726 (1.533–1.944)	*p* < 0.001
Radiation	29.3%	5.9%	7.5 (6.6–8.4)		Reference	
Chemotherapy	28.7%	7.2%	6.4 (4.6–8.3)		1.409 (1.103–1.799)	*p* = 0.006
Chemoradiotherapy	58.2%	10.7%	13.6 (13.3–13.8)		0.684 (0.615–0.760)	*p* < 0.001

Abbreviations: OS, overall survival; CI, confidence interval

^1^Adjusted for age, sex, race, ethnicity, Charlson/Deyo comorbidity score, income, education, geographic region, insurance status, facility type, facility location, extent of resection, and treatment regimen

^*^Variables with statistical significance are shown in bold

### Overall survival and mortality risk after stratifying patients by age

Among the elderly, patients undergoing GTR demonstrated superior 1-year and 3-year OS (43.8% and 11.0%) relative to patients undergoing STR (30.3% and 8.0%) or biopsy (15.1% and 2.4%) (Supplementary Table S2). Extent of resection was also significantly associated with OS in the elderly *MGMT*-methylated and *MGMT*-unmethylated cohorts (both *p* < 0.001). Among elderly patients with *MGMT*-unmethylated tumors, the median OS was 6.2 months with RT alone and 4.3 months with CT alone. Among elderly patients with *MGMT*-methylated tumors, however, the median OS was 4.4 months with RT alone and 5.7 months with CT alone. After adjusting for baseline sociodemographic and clinical characteristics, CT alone was associated with an increased risk of mortality (HR 1.63, 95% CI 1.09–2.44) compared to RT alone in elderly patients with *MGMT*-unmethylated tumors, while CT alone was associated with a decreased risk of mortality (HR 0.71, 95% CI 0.51–0.99) compared to RT alone in elderly patients with *MGMT*-methylated tumors. CRT use was associated with longer median OS and a lower risk of death compared to RT alone in elderly patients with *MGMT*-methylated (13.7 vs. 4.4 months; HR 0.39, 95% CI 0.32–0.49) and *MGMT*-unmethylated tumors (10.2 vs. 6.2 months; HR 0.77, 95% CI 0.66–0.90). The survival outcomes and mortality risk for patients < 70 years stratified by extent of resection and treatment regimen are shown in Supplementary Table S3.

## Discussion

In this retrospective analysis of the NCDB, a high proportion of patients recently diagnosed with GBM in the United States underwent *MGMT* testing (74.6%). The median OS of the entire cohort was 12.4 months (16.4 months for *MGMT*-methylated patients and 11.8 months for *MGMT*-unmethylated patients). Although there was a decline in CRT use with increasing age, RT alone was more commonly administered than CT alone for elderly patients with *MGMT*-unmethylated tumors (11.2% vs. 2.1%) and *MGMT*-methylated tumors (6.6% vs. 3.9%). However, for elderly patients with *MGMT*-methylated tumors, CT alone was associated with a lower risk of mortality compared to RT alone. In contrast, for elderly patients with *MGMT*-unmethylated tumors, CT alone was associated with a higher risk of mortality compared to RT alone.

The proportion of patients receiving *MGMT* testing in our cohort (74.6%) is a significant increase over the proportion reported by Lee et al. (13%), who analyzed data from the 2010–2012 NCDB among patients with histologically-diagnosed GBM [17]. This likely reflects the incorporation of *MGMT* testing into standardized NCCN guidelines for high grade gliomas in 2013. Although *MGMT* promoter methylation status is not used for treatment stratification in non-elderly patients (< 70 years) with good functional status [12], *MGMT* testing is still valuable as a prognostic marker [13]. Among non-elderly patients in our cohort receiving CRT, the median OS was 24.3 months for *MGMT*-methylated patients and 14.6 months for *MGMT*-unmethylated patients. These results are comparable to the median OS for methylated patients (23.4 months) and unmethylated patients (12.6 months) treated with TMZ and RT in the Stupp trial [6]. We should note, however, that patients in our cohort had access to newer treatments. The addition of tumor-treating fields to maintenance TMZ has resulted in improved median OS (20.9 vs. 16.0 months, *p* < 0.001) compared to maintenance TMZ alone in patients with newly diagnosed GBM [18], and approximately 30% of eligible GBM patients receive tumor-treating fields in countries where the therapy is available [19–20].

De-escalation of combined CRT, guided by *MGMT* methylation status, is important among elderly patients, or patients with poor functional status, who cannot tolerate multimodal therapy [8–9, 21]. Our findings that CT alone is associated with a lower risk of death compared to RT alone for elderly patients with methylated tumors, while CT alone is associated with a higher risk of death compared to RT alone for elderly patients with unmethylated tumors match results from a meta-analysis that included both the Nordic and NOA-08 trials as well as three additional comparative studies (*n* = 973 patients) [10, 22, 23]. Notably, elderly patients in our cohort were more likely to receive RT alone than CT alone regardless of *MGMT* promoter methylation status. While elderly GBM patients receiving TMZ or RT demonstrate similar quality of life, treatment with TMZ is associated with a higher incidence of lymphocytopenia, neutropenia, infection, thromboembolism, and abnormal liver enzymes [10, 22]. Moreover, CT toxicity is strongly correlated with increasing age [24]. Therefore, it is plausible that patient preferences for fewer side effects guide treatment selection later in life.

Several barriers preclude the implementation of *MGMT* promoter methylation status into routine clinical decision-making. An approximate 85% rate of concordance is observed between *MGMT* promoter methylation and mRNA expression [25]. Therefore, a considerable minority of unmethylated patients with low expression of *MGMT* may potentially be sensitive to TMZ. However, there is ongoing re-evaluation of the degree of *MGMT* methylation, as some assays may be able to discern between unmethylated and “truly unmethylated” *MGMT* gene promoters [26]. Moreover, in a separate analysis containing only patients with known *MGMT* promoter methylation status from the landmark Stupp trial in 2005, the combination of RT and TMZ did not significantly prolong survival compared to RT alone for patients with *MGMT*-unmethylated tumors (12.7 vs. 11.8 months, *p* = 0.06), but did prolong survival for patients with *MGMT*-methylated tumors (21.7 vs. 15.3 months, *p* = 0.007) [5]. Similarly, Perry et al. showed that the addition of TMZ to hypofractionated RT (40 Gy in 15 fractions) did improve survival for elderly patients with *MGMT*-methylated tumors (13.5 vs. 7.7 months, *p* < 0.001), but did not improve survival for elderly patients with *MGMT*-unmethylated tumors (10.0 vs. 7.9 months, *p* = 0.055) [27]. Despite lack of statistical significance in both of these trials for patients with unmethylated tumors, there was a clinical benefit with CRT, which raised the question of whether TMZ should be administered to all GBM patients regardless of *MGMT* promoter methylation status.

While CRT was associated with better survival than RT alone for patients in our cohort irrespective of *MGMT* promoter methylation status, the NCDB does not contain Karnofsky Performance Status (KPS) scores. Current SNO and EANO guidelines recommend RT or CT alone only for patients with poor prognostic factors (i.e., increasing age and/or low KPS score) [28]. Therefore, patients with poorer baseline health in our cohort likely received treatment with one modality, confounding our analysis. Furthermore, while there was a decrease in CRT use with increasing age, only a minority of elderly patients in our cohort received either CT or RT alone (< 12%). Nearly 30% of elderly patients in our study did not receive CT or RT, which is surprising given that CT or RT monotherapy has been shown to confer longer survival than supportive care alone in elderly GBM patients [29–30]. This suggests that a large proportion of elderly patients are being deemed ineligible for treatment, opting to forgo treatment, or struggling to overcome barriers to oncologic care.

Our study also illuminates the areas where *MGMT* testing is underutilized, which may provide a focused starting point for improving access to this aspect of care for GBM. For example, increasing age was associated with a lower likelihood of receiving *MGMT* testing, which is problematic as elderly patients are generally more susceptible to the toxic side effects of combined CRT [30]. A recent reanalysis of the Nordic, NOA-08, and CE.6 trials proposes to drop TMZ when treating truly *MGMT*-unmethylated GBM patients in the elderly [31]. An increasing number of randomized trials have also started withholding TMZ in *MGMT*-unmethylated patients in favor of alternative treatment without detrimental effects on survival outcomes [8, 32–33].

Treatment at non-academic programs was also associated with lower odds of receiving *MGMT* testing in our cohort. Several methods to test for *MGMT* promoter methylation include direct bisulfite sequencing, methylation-specific polymerase chain reaction, and pyrosequencing, among others [3]. Pyrosequencing is generally regarded as more accurate than other tests, but it is also significantly more expensive [34]. Non-academic facilities may not have the equipment or personnel required for *MGMT* testing, and the turnaround time for obtaining the test at an outside institution may deter providers from ordering the test. Additionally, since *MGMT* testing is not required for the diagnosis of GBM according to the 2021 World Health Organization criteria [35], insurance companies may not cover the cost of testing [36].

To address these disparities, future clinical guidelines should emphasize routine *MGMT* testing for all newly diagnosed GBM patients, highlighting populations that are less likely to undergo molecular testing such as the elderly, those with limited insurance coverage, and communities with lower educational attainment. With an increasing number of clinical trials using *MGMT* testing as an enrollment criterion, expanding insurance coverage or providing subsidies for testing could improve accessibility for all patients. Additionally, reforms at the hospital level could be implemented to ensure adequate infrastructure for timely *MGMT* testing. Taken together, *MGMT* testing can provide crucial insights into optimal treatment options for GBM patients, in addition to acting as a prognostic marker, and should be considered standard practice in the comprehensive management of GBM.

## Limitations

Limitations of the present study include the retrospective nature of data collection. We also excluded patients with unknown *IDH*-status. Data from the 2018–2020 Central Brain Tumor Registry of the United States, which represents nearly 100% of the U.S. population, showed 16.4% of glioblastoma histology had unknown *IDH* status [1]. Since molecular testing was not standard practice during the study period, a significant number of patients were likely excluded, which results in sampling bias. It is also plausible that patients who opted for *IDH*-mutation testing were more likely to opt for *MGMT* testing. We also could not delineate the method of *MGMT* testing or the timing relative to diagnosis. Other pertinent information including specific chemotherapeutic regimens were missing from the NCDB. Currently, the NCDB lists over 600 antineoplastic agents as chemotherapy. Therefore, the proportion of patients receiving CT alone with TMZ is likely lower than the percentages reported in this study. Another limitation of this study is the absence of data on KPS scores, which are an important factor in treatment selection for GBM patients. For patients with KPS scores < 60, the risks of treatment-related toxicity may outweigh the benefits, and palliative or best supportive care may be prioritized over RT and/or CT [8–9]. We also could not discern treatment patterns based on patient preferences, or patient-physician shared decision-making, as this data is not captured in the NCDB. We were also not able to verify patients that were prescribed tumor-treating fields or enrolled in clinical trials due to potential underreporting by the NCDB.

## Conclusion

Despite NCCN guidelines emphasizing the incorporation of tumor molecular analysis, a substantial proportion of GBM patients in the United States still do not undergo *MGMT* promoter testing. This valuable prognostic and predictive marker may help to determine optimal treatment in the elderly that is supported by population-specific clinical trials. Our results demonstrate that while there was a decrease in CRT use with increasing age, RT alone was more commonly administered to elderly patients than CT alone, irrespective of *MGMT* promoter methylation status. Increased utilization of *MGMT* promoter methylation testing to guide treatment decisions may improve quality of life and survival for elderly patients.

## Electronic supplementary material

Below is the link to the electronic supplementary material.

Supplementary Material 1

## Data Availability

Researchers may request NCDB data from the American College of Surgeons and the American Cancer Society. Requests to access the datasets should be directed to https://www.facs.org/quality-programs/cancer-programs/national-cancer-database/puf/.
