# Supplementary material for: Treatment practices and survival outcomes for IDH-wildtype glioblastoma patients according to MGMT promoter methylation status: insights from the U.S. National Cancer Database
Source: J Neurooncol. 2025 Feb 5;172(3):655–65. doi: 10.1007/s11060-025-04952-y (PMC11968476; doi:10.1007/s11060-025-04952-y)
Supplement: Supplementary file 1 — Supplementary Material 1 [file 11060_2025_4952_MOESM1_ESM.docx]

**Supplementary Table S1**. Coding definitions for methylation status of the O^6^-methylguanine-DNA methyltransferase (*MGMT*) gene promoter.

| Code | Description |
| --- | --- |
| 0 | *MGMT* methylation absent/not present, unmethylated *MGMT* |
| 1 | *MGMT* methylation present, low level Hypomethylated Partial methylated |
| 2 | *MGMT* methylation present, high level Hypermethylated |
| 3 | *MGMT* methylation present, level unspecified |
| 7 | Test ordered, results not in chart |
| 8 | Not applicable: Information not collected for this case |
| 9 | Not documented in patient record Cannot be determined by the pathologist *MGMT* not assessed or unknown if assessed |

The content of this table was adapted from the North American Association of Central Cancer Registries (NAACCR) site-specific data item #3889.

**Supplementary Table S2**. Survival analyses of patients ≥70 years diagnosed with glioblastoma, *IDH*-wildtype stratified by *MGMT* promoter methylation status.

|  | 1-year OS | 3-year OS | Median OS (95% CI) | p-value^*^ | Hazard Ratio (95% CI)^1^ | p-value^*^ |
| --- | --- | --- | --- | --- | --- | --- |
|  |  |  |  |  |  |  |
| **Entire Age ≥70 Cohort** | 31.8% | 7.9% | 6.8 (6.5-7.1) |  |  |  |
|  |  |  |  |  |  |  |
| Extent of Resection |  |  |  | **p<0.001** |  | **p<0.001** |
| Biopsy | 15.1% | 2.4% | 3.4 (3.1-3.6) |  | Reference |  |
| Subtotal | 30.3% | 8.0% | 6.8 (6.3-7.2) |  | 0.655 (0.605-0.709) | p<0.001 |
| Gross total | 43.8% | 11.0% | 10.2 (9.6-10.8) |  | 0.527 (0.486-0.573) | p<0.001 |
|  |  |  |  |  |  |  |
| Treatment Regimen |  |  |  | **p<0.001** |  | **p<0.001** |
| No radiation or chemotherapy | 9.7% | 4.3% | 2.1 (1.9-2.2) |  | 1.868 (1.668-2.092) | p<0.001 |
| Radiation | 19.2% | 4.8% | 5.2 (4.6-5.8) |  | Reference |  |
| Chemotherapy | 17.2% | 4.5% | 5.0 (3.9-6.1) |  | 0.950 (0.771-1.170) | p=0.627 |
| Chemoradiotherapy | 45.1% | 10.3% | 10.9 (10.5-11.3) |  | 0.586 (0.526-0.652) | p<0.001 |
|  |  |  |  |  |  |  |
| ***MGMT* Methylated** | 39.9% | 11.9% | 8.2 (7.5-8.9) |  |  |  |
|  |  |  |  |  |  |  |
| Extent of Resection |  |  |  | **p<0.001** |  | **p<0.001** |
| Biopsy | 20.3% | 3.7% | 3.8 (3.2-4.4) |  | Reference |  |
| Subtotal | 39.0% | 12.5% | 8.3 (7.3-9.4) |  | 0.621 (0.538-0.717) | p<0.001 |
| Gross total | 52.1% | 16.1% | 12.9 (11.3-14.4) |  | 0.521 (0.448-0.606) | p<0.001 |
|  |  |  |  |  |  |  |
| Treatment Regimen |  |  |  | **p<0.001** |  | **p<0.001** |
| No radiation or chemotherapy | 10.8% | 5.2% | 2.2 (2.0-2.4) |  | 1.487 (1.191-1.857) | p<0.001 |
| Radiation | 13.7% | 6.4% | 4.4 (3.7-5.1) |  | Reference |  |
| Chemotherapy | 18.9% | 5.4% | 5.7 (3.7-7.6) |  | 0.706 (0.506-0.985) | p=0.041 |
| Chemoradiotherapy | 55.1% | 15.5% | 13.7 (12.7-14.8) |  | 0.393 (0.318-0.486) | p<0.001 |
|  |  |  |  |  |  |  |
| ***MGMT* Unmethylated** | 29.0% | 5.0% | 7.2 (6.7-7.6) |  |  |  |
|  |  |  |  |  |  |  |
| Extent of Resection |  |  |  | **p<0.001** |  | **p<0.001** |
| Biopsy | 12.4% | 2.4% | 3.6 (3.2-4.0) |  | Reference |  |
| Subtotal | 24.2% | 4.3% | 6.4 (5.8-6.9) |  | 0.736 (0.643-0.843) | p<0.001 |
| Gross total | 41.8% | 6.9% | 10.1 (9.4-10.7) |  | 0.549 (0.477-0.631) | p<0.001 |
|  |  |  |  |  |  |  |
| Treatment Regimen |  |  |  | **p<0.001** |  | **p<0.001** |
| No radiation or chemotherapy | 9.3% | 3.8% | 2.2 (1.9-2.4) |  | 2.305 (1.948-2.728) | p<0.001 |
| Radiation | 24.5% | 6.4% | 6.2 (5.1-7.4) |  | Reference |  |
| Chemotherapy | 11.2% |  | 4.3 (3.2-5.4) |  | 1.634 (1.094-2.441) | p=0.017 |
| Chemoradiotherapy | 39.5% | 5.7% | 10.2 (9.7-10.6) |  | 0.773 (0.661-0.903) | p=0.001 |

Abbreviations: OS, overall survival; CI, confidence interval.

^1^Adjusted for age, sex, race, ethnicity, Charlson/Deyo comorbidity score, income, education, geographic region, insurance status, facility type, facility location, extent of resection, and treatment regimen.

^*^Variables with statistical significance are shown in bold.

Cell left blank could not be calculated due to insufficient follow-up.

**Supplementary Table S3.** Survival analyses of patients <70 years diagnosed with glioblastoma, *IDH*-wildtype stratified by *MGMT* promoter methylation status

|  | 1-year OS | 3-year OS | Median OS (95% CI) | p-value^*^ | Hazard Ratio (95% CI)^1^ | p-value^*^ |
| --- | --- | --- | --- | --- | --- | --- |
|  |  |  |  |  |  |  |
| **Entire Age <70 Cohort** | 59.9% | 18.8% | 14.9 (14.7-15.2) |  |  |  |
|  |  |  |  |  |  |  |
| Extent of Resection |  |  |  | **p<0.001** |  | **p<0.001** |
| Biopsy | 34.7% | 8.6% | 7.4 (6.9-7.9) |  | Reference |  |
| Subtotal | 58.0% | 17.3% | 14.1 (13.7-14.5) |  | 0.604 (0.566-0.644) | p<0.001 |
| Gross total | 70.1% | 23.6% | 17.9 (17.5-18.4) |  | 0.462 (0.433-0.493) | p<0.001 |
|  |  |  |  |  |  |  |
| Treatment Regimen |  |  |  | **p<0.001** |  | **p<0.001** |
| No radiation or chemotherapy | 23.1% | 9.6% | 2.9 (2.7-3.1) |  | 1.550 (1.374-1.748) | p<0.001 |
| Radiation | 37.8% | 8.5% | 9.3 (8.2-10.4) |  | Reference |  |
| Chemotherapy | 44.4% | 14.0% | 9.8 (7.4-12.2) |  | 0.877 (0.725-1.059) | p=0.172 |
| Chemoradiotherapy | 67.9% | 20.9% | 16.9 (16.5-17.2) |  | 0.528 (0.474-0.590) | p<0.001 |
|  |  |  |  |  |  |  |
| ***MGMT* Methylated** | 68.7% | 29.8% | 21.0 (20.1-21.8) |  |  |  |
|  |  |  |  |  |  |  |
| Extent of Resection |  |  |  | **p<0.001** |  | **p<0.001** |
| Biopsy | 45.3% | 11.1% | 9.6 (7.8-11.4) |  | Reference |  |
| Subtotal | 65.9% | 27.3% | 19.8 (18.6-21.1) |  | 0.573 (0.504-0.651) | p<0.001 |
| Gross total | 78.4% | 37.5% | 26.9 (25.3-28.5) |  | 0.434 (0.381-0.494) | p<0.001 |
|  |  |  |  |  |  |  |
| Treatment Regimen |  |  |  | **p<0.001** |  | **p<0.001** |
| No radiation or chemotherapy | 20.9% | 9.6% | 2.8 (2.4-3.2) |  | 1.952 (1.496-2.547) | p<0.001 |
| Radiation | 45.3% | 10.8% | 10.7 (6.7-14.8) |  | Reference |  |
| Chemotherapy | 44.1% | 14.8% | 9.8 (6.8-12.7) |  | 0.914 (0.631-1.322) | p=0.632 |
| Chemoradiotherapy | 76.7% | 33.3% | 24.3 (23.3-25.2) |  | 0.484 (0.378-0.620) | p<0.001 |
|  |  |  |  |  |  |  |
| ***MGMT* Unmethylated** | 56.8% | 11.7% | 13.5 (13.2-13.7) |  |  |  |
|  |  |  |  |  |  |  |
| Extent of Resection |  |  |  | **p<0.001** |  | **p<0.001** |
| Biopsy | 30.6% | 6.0% | 7.3 (6.7-8.0) |  | Reference |  |
| Subtotal | 54.1% | 9.9% | 12.9 (12.5-13.2) |  | 0.642 (0.582-0.709) | p<0.001 |
| Gross total | 66.7% | 15.0% | 15.6 (15.2-16.0) |  | 0.472 (0.428-0.522) | p<0.001 |
|  |  |  |  |  |  |  |
| Treatment Regimen |  |  |  | **p<0.001** |  | **p<0.001** |
| No radiation or chemotherapy | 24.7% | 9.8% | 3.0 (2.6-3.4) |  | 1.263 (1.065-1.498) | p=0.007 |
| Radiation | 33.4% | 6.0% | 9.0 (7.7-10.3) |  | Reference |  |
| Chemotherapy | 38.7% | 9.9% | 8.3 (4.5-12.2) |  | 1.115 (0.814-1.526) | p=0.498 |
| Chemoradiotherapy | 63.4% | 12.1% | 14.6 (14.3-14.9) |  | 0.577 (0.497-0.669) | p<0.001 |

Abbreviations: OS, overall survival; CI, confidence interval.

^1^Adjusted for age, sex, race, ethnicity, Charlson/Deyo comorbidity score, income, education, geographic region, insurance status, facility type, facility location, extent of resection, and treatment regimen.

^*^Variables with statistical significance are shown in bold.
